# A qualitative study of pharmacy nurse providers of community based post-birth care in Queensland, Australia

**DOI:** 10.1186/1471-2393-13-144

**Published:** 2013-07-10

**Authors:** Maria Zadoroznyj, Wendy Brodribb, Lauren Falconer, Lauren Pearce, Casey Northam, Sue Kruske

**Affiliations:** 1 Institute for Social Science Research and School of Social Science, The University of Queensland, Brisbane, Queensland, Australia; 2 Discipline of General Practice, School of Medicine, The University of Queensland, Brisbane, Queensland, Australia; 3 School of Social Science, The University of Queensland, Brisbane, Queensland, Australia; 4 Queensland Centre for Mothers & Babies, School of Psychology, The University of Queensland, Brisbane, Queensland, Australia

**Keywords:** Postnatal care, Pharmacy nurse, Child health clinic

## Abstract

**Background:**

Reduced length of hospital stay following childbirth has placed increasing demands on community-based post-birth care services in Australia. Queensland is one of several states in Australia in which nurses are employed privately by pharmacies to provide maternal and child health care, yet little is known about their prevalence, attributes or role. The aims of this paper are to (1) explore the experiences and perspectives of a sample of pharmacy nurses and GPs who provide maternal and child health services in Queensland, Australia (2) describe the professional qualifications of the sample of pharmacy nurses, and (3) describe and analyze the location of pharmacy nurse clinics in relation to publicly provided services.

**Methods:**

As part of a state-wide evaluation of post-birth care in Queensland, Australia, case studies were conducted in six regional and metropolitan areas which included interviews with 47 key informants involved in postnatal care provision. We report on the prevalence of pharmacy nurses in the case study sites, and on the key informant interviews with 19 pharmacy nurses and six General Practitioners (GPs). The interviews were transcribed and analysed thematically.

**Results:**

The prevalence of pharmacy nurses appears to be highest where public services are least well integrated, coordinated and/or accessible. Pharmacy nurses report high levels of demand for their services, which they argue fill a number of gaps in the public provision of maternal and child health care including accessibility, continuity of carer, flexibility and convenient location. The concerns of pharmacy nurses include lack of privacy for consultations, limited capacity for client record keeping and follow up, and little opportunity for professional development, while GPs expressed concerns about inadequate public care and about the lack of regulation of pharmacy based care.

**Conclusions:**

Pharmacy based clinics are a market-driven response to gaps in the public provision of care. Currently there are no minimum standards or qualifications required of pharmacy nurses, no oversight or regulation of their practice, and no formal mechanisms for communicating with other providers of postnatal care. We discuss the implications and possible mechanisms to enhance best-practice care.

## Background

Australian women spend an average of two days in hospital after a non-instrumental vaginal birth and four days in hospital after a lower segment caesarean section, and mothers in the state of Queensland have the highest rate of hospital discharge before five days in Australia [1]. Short postnatal hospital stays mean that care and education following childbirth must increasingly take place in the community. While Australia has a well-established system of universal service provision to meet the primary health care needs of new mothers and their babies, recent evidence indicates that the delivery of primary care services are “inconsistent across jurisdictions, fragmented across disciplines and sectors, and currently do not adequately meet the needs of the population” [2].

In many Australian states including Queensland, the professional groups providing post-discharge care to new mothers and their babies include domiciliary midwives (usually employed by hospitals), Child and Family Health Nurses (CFHNs) typically employed in community Child and Family Health Centres (CFHCs), and General Practitioners (GPs) [3-5]. Child and family health nurses and domiciliary midwives are generally employed in the public health sector and provide maternal and infant health services that are free of charge, while GP services may involve some out-of-pocket fees. Queensland is one of several states in Australia where, in addition to publicly provided services, pharmacies employ nurses to provide maternal and child health care in retail pharmacy outlets [6]. In other domains of primary care provision, nurses based in pharmacies are also providing support to those who transition from acute to community settings [7-10]. While such services may improve access to care, little is known about their implications for quality of care, or their implications for the integration and coordination of health care services overall – key elements of Australia’s Primary Health Care Strategy [11,12]. The pharmacy nursing model of care is unique to Australia. It is similar in purpose to well-baby services in North America, however those services are delivered on a fee-for-service basis and are provided or overseen by physicians in clinical settings [13]. Similar again is the Health Visiting Service in the United Kingdom, which is delivered by nurses but is publically funded and occurs in the homes of women with newborns for a fixed period of time after birth [14]. It is also distinct from Nurse Practitioners’ clinics, which are a relatively new development in both Australia [15] and North America [16], in that pharmacy nurses are not necessarily nurse practitioners, services are not offered on a fee-for-service basis and there is no requirement for formal collaboration between pharmacy nurses and medical practitioners. The pharmacy nursing model of care considered here is perhaps most similar to the increasingly frequent examples, both within Australia and internationally, of pharmacies providing additional services, such as bone-density screening [17] or minor ailment schemes [18].

There is considerable evidence that currently primary care in Australia lacks integration and coordination [11]. This is a particular problem for new mothers following discharge from hospital or birthing facility, as there is wide variation in how follow up care for new mothers and their infants is arranged across health districts and States and Territories in Australia [2,5]. As a result, new mothers in Australia report high levels of dissatisfaction with community based postnatal care [3,19-21].

In Queensland, an independent review of maternity services reported consumer concerns about the lack of postnatal support and difficulty accessing child health centres [22]. A key recommendation of this review was to improve the provision of postnatal care [22], and in response the Queensland Government funded the Universal Postnatal Contact Service (UPNCS), which was rolled out between 2008 and 2010 [4]. The UPNCS ensured, amongst other things, at least one telephone call or home visit by a health care provider post-discharge to new mothers who had given birth in a public birthing facility.

In 2011, the UPNCS program in Queensland was evaluated, and in this paper we draw on data from the state-wide evaluation of the program. The evaluation included the identification and examination of post-discharge health care services already available to mothers and families at the time of the implementation of UPNCS. In this paper, we focus on the nurses who practise in, and are employed by pharmacies to provide maternal and child health care. These nurses have become important contributors to maternal and child health care in the community in Queensland, yet, with the exception of one study [6] little is known about their prevalence, their professional attributes and motivations, or their working relationships with other public or private primary care providers of maternal and child health services. In this paper, we address this gap in existing research. The aims of this paper are to: (1) explore the experiences and perspectives of a sample of pharmacy nurses and GPs on the genesis and nature of the role of pharmacy nurses in the provision of maternal and child health services in Queensland, Australia, (2) to describe the professional attributes of pharmacy nurses, and (3) to describe and analyze the prevalence of pharmacy clinics in relation to publicly provided services. We consider the implications of the current practice of pharmacy employed nurses for issues of access, quality of care, and the coordination and integration of post-birth care in the community.

## Methods

The data analysed in this paper were collected as part of a state-wide evaluation of UPNCS [4]. The evaluation included a comparative case study of six sites in rural and regional parts of the state, as well as a major metropolitan centre. Researchers conducted face-to-face interviews about postnatal care with 117 stakeholders in total, observed facilities and collected informational material and relevant documents [4]. Pharmacy nurses, child health nurses and GPs were interviewed as part of the key stakeholder groups providing community based care to mothers and their infants. In this paper we draw on the interviews with 19 pharmacy nurses for their perspectives on the genesis and nature of their role, and on the perspectives of six GPs known to have an interest in postpartum care in the case study sites.

### Recruitment of sample

In the non-metropolitan sites with pharmacy nurse clinics, we contacted pharmacies employing nurses directly by telephone. The study was described to the pharmacy manager or equivalent, who was then asked to pass on details of the study and researcher contact details to the pharmacy nurses. Pharmacy nurses were asked to call us if they wished to participate in the study. Both of the two pharmacy nurses in one site, and three of six in the other regional case study site were recruited to the study.

A more complex sampling and recruitment strategy was required for the city of Brisbane. Brisbane is the capital of Queensland, with a population of 1,079,392 within the Brisbane City Council (BCC) local government area in June 2011. Prior to this research, no systematic attempt had been made to enumerate the proportion of pharmacies offering a ‘pharmacy nurse’ baby clinic within the city, nor to map their geographic distribution. As a first step in building an appropriate information base, researchers mapped the distribution of all pharmacies in the BCC area through listings in http://www.yellowpages.com.au and http://www.truelocal.com.au. Secondly, all 301 pharmacies listed in the BCC catchment area were telephoned, and asked whether they employed a pharmacy nurse to provide a ‘baby clinic’. Of the 56 pharmacies (18.6%) employing a pharmacy nurse, 40 were randomly selected and contacted by telephone to request participation in the study, and 14 participants were recruited in the Brisbane city area. In total 19 pharmacy nurses working in city and regional centres were interviewed, primarily through face-to-face interviews (*N* = *15*), although 4 nurses were interviewed by telephone or completed an online survey.

GPs with an interest in postpartum care were purposefully recruited through contacts of one of the research team.

Semi-structured interviews lasting between half an hour and an hour were conducted at mutually convenient locations, including pharmacies, GP clinics and other public venues. Respondents were asked about a variety of aspects of their work experience, their credentials and their perceptions of their roles. A summary of the topic guide can be provided by request to the author. Following consent, interviews were audio-recorded and transcribed verbatim.

In addition to the qualitative interviews, researchers also mapped the prevalence and geographic distribution of pharmacy clinics with publicly provided CFHCs in the case study sites. In the BCC, mapping compared the accessibility and availability of pharmacy clinics with CFHCs using information publicly available through the Queensland Health website. Opportunistic observation of 10 of the pharmacies where pharmacy nurses worked took place at the time of interview.

The majority of data collection took place between June and November 2011, although some interviews took place between Jan 2011 and June 2012.

### Data analysis

Qualitative data gathered through interviews were analysed using thematic analysis with *Nvivo 9* software. Coding of data was initially conducted independently by several of the researchers involved in data collection; subsequently the themes identified were further explored in consultation, with high degree of consensus regarding the reliability of thematic interpretations [23].

#### Ethical considerations

Before conducting the research, ethical approval was obtained by the University of Queensland (UQ), and the UQ School of Social Science Ethical Review Panels. Informed consent was obtained from participants. To ensure anonymity and confidentiality all data were kept securely in password locked computer files and all information gathered in interviews was de-identified in the transcripts.

## Results

### The attributes and qualifications of pharmacy nurses

Our all-female sample were all registered nurses (*N* = *19*), and most (*N* = *16*) had qualifications in midwifery, while a significant proportion were also credentialed in child health (*N* = *10*). Only a minority were qualified as lactation consultants (*N* = *4*) with a few holding other relevant qualifications such as paediatric nursing or child development. All had experience in providing care to mothers and babies prior to working in the pharmacy. Most recounted extensive work histories of 15 years or more in nursing, and most had practised primarily in midwifery and child health. Seventeen of the 19 respondents worked in the pharmacy for less than 15 hours per week, with six to ten hours per week the modal category, generally in one or two clinics of three to four hours duration per week. Almost all interviewees held one other part-time job as a nurse in a hospital or General Practice clinic. In the pharmacies they were employed as part-time or casual staff on hourly pay rates. Our respondents, especially those caring for families, were attracted to work in the clinics because of the predictable, part-time work within school hours.

“…*the hours here are very suitable here for me and my family.*” RESP 6

“*Before I became a pharmacy nurse*, *I used to work for Child Health as a Child Health Nurse, and then I had my own children and the hours in the Child Health service were very inflexible so I really couldn’t work you know like just four hours a day sort of thing, so the pharmacy allowed me to do that.*” RESP 11

This well qualified and experienced group of nurses provided considerable insight into their motivations, duties, sources of job satisfaction, working relationships with other primary care providers and concerns with their work. In the next part of the paper, we report on the major themes that emerged from the interviews with pharmacy nurses and GPs. These included the strong sense that the work of pharmacy nurses fills a gap in the public provision of maternal and child health services. Pharmacy nurses felt that they provided an accessible, convenient service and that they could provide flexible, responsive care. They were also able to articulate a number of limitations and concerns about their conditions of practice.

### The genesis of pharmacy nursing in Queensland

“Filling a gap”

For both the GPs and the pharmacy nurses in our sample, the lack of sufficient public CFHC services was a significant problem, which contributed to the genesis of pharmacy nurse clinics. For example, one of the GPs in our sample said:

“*..one of the reasons for not referring to early childhood nurses [CFHNs] more is there just isn’t capacity.....Fund it properly… If I said to all of my new mums go along and meet the early childhood nurses, talk to them about breastfeeding…the unsettled baby…get your baby weighed and measured every week for the first six weeks, they would be overwhelmed, they can’t actually deliver, so all I do is set women up with an unrealistic expectation on an overloaded and already stretched service…”* GPBR1

The pharmacy nurses themselves overwhelmingly described their services as ‘filling a gap’ in the public provision of care in the context of the retrenchment of public services. While one of our respondents simply indicated that pharmacy nurse clinics:

“ *fill a gap that is not provided by the public system.”* RESP 4

Others elaborated extensively, for example:

*“I just think that pharmacy nurses probably evolved because of the poor quality of the child health service that was there for women, and that there was a need in the community for women to be able to have easy to access Child Health Nurses. When I started doing Child Health twenty years ago there were centres in every suburb, women found it very accessible they’d come every week, and then … closed them down … and eventually they only had big major centres ……so people would have to drive there … so they’ve actually closed so many of them now that women find it really difficult to access them there is obviously now a big wait for them too, like four weeks to get in and so I think out of that evolved pharmacy nurses.”* RESP 11

“*Thirty years ago there were so many government provided clinics, a mum could almost walk in at any time and get helped. But the government has moved away from that type of provision.”* RESP 9

The nurses were well aware that publicly provided services have become increasingly targeted towards ‘at-risk’ mothers, with the result that sometimes “…*normal mums get forgotten”* RESP 9.

One of the nurses put this in terms of a ‘zero-sum’ situation:

*“There used to be child health, equivalent down the street. I used to work there myself… [now] it’s not funded because all of the funding goes into the at risk mothers who are in the community and get home visited.”* RESP 5

Our examination of the geographic distribution and availability of pharmacy nurse clinics and publicly provided CFHCs concurs with the notion that pharmacy nurses ‘fill a gap’ created by the retrenchment and re-orientation (to ‘targeting’) of publicly provided maternal and child health services. Across our case study sites, for example, the number and distribution of pharmacies employing nurses varied widely. Two of the four regional and rural sites which had reasonably well integrated and comprehensive community based maternal and child health services, did not have pharmacy nurses at all, whereas one of the case study sites in which post-hospital discharge services were poorly integrated and coordinated, two pharmacy nurses provided clinics at four different pharmacies. In the BCC area, we identified a large disparity in the prevalence of pharmacy nurse clinics (n = 56), compared with CFHCs (n = 13). There were other significant differences in availability and access between the two types of clinics. Seven of the 13 CFHCs were open every weekday, and the remainder open once or twice per week; most of the public clinics operated on an appointment only basis with average waiting times of between 2 and 4 weeks for an appointment. In contrast, the larger number of pharmacy clinics were open shorter hours (generally open for half or whole days once or twice per week), but almost all were available on a drop-in basis, so parents did not have to wait for an appointment.

### Quality of service provision: responsive, flexible, continuity of carer

In addition to ‘filling a gap’, pharmacy nurses also emphasised a number of key features which distinguished their work from CFHNs working in public CFHCs. As mentioned, most of the pharmacy nurses ran their clinics on a ‘drop in’ rather than appointment basis, and hence perceived their services as more responsive to client needs and demands than public (primarily appointment only) centres.

Pharmacy nurses, many of whom had previously worked in CFHCs, perceived their practice as more autonomous and flexible than nurses working in CFHCs. They described their work in pharmacies as free of the bureaucratic and organisational constraints governing the practice of CFHNs. They also articulated their capacity to work without oversight from their employers and hence to be able to practice autonomously.

*“I probably think that [public] Child Health is very inflexible in that it seems to go a lot on what books say.”* RESP 11

In relation to infant feeding, a number of pharmacy nurses talked about their capacity to support the mother in her decisions, rather than ‘rigidly’ promoting breast feeding, for example:

“….*if a woman walks in and says I'm giving my baby a bottle - … I [don’t] say to her ‘you're doing the wrong thing’ - I like that it's more flexible* [*here than at CFHC]. I'm aware of the [promotion of breastfeeding] guidelines, I support them but you're dealing with real people. I'm allowed to do … that. I get the reverse of it where people come to me and say, ‘they're too pushy [at CFHC] and I like you because you're not”*. RESPR1

Pharmacy nurses were positive about their capacity to respond to the individual needs of their clients and provide ‘woman centred’ care:

*“I just say, I'm available as much or as little as you want to come - whatever you feel you need.”* RESPR1

The pharmacy nurses in our sample also valued the fact that they provided continuity of care by virtue of being the one care provider in a pharmacy clinic. They indicated that most of the parents they saw were ‘regulars’, and they spoke of this continuity as an intrinsic source of job satisfaction:

*“…enormous satisfaction, particularly when I have parents coming back with second and third babies.”* RESP 7

Another said:

*“I've been here for ten years and I'll see people that have had four kids so you know all their kids.”* RESPR1

This kind of continuity enabled pharmacy nurses to provide personalised care, even when ‘off duty’ as the following shows:

*“But I'll tell them if they're having problems and they need to, I am over here on Tuesday if you need me. They can ring me up here. If I know them and I'm really comfortable, I'll tell them they can ring me at home if they're really stuck.”* RESPR1

Most pharmacy clinics are located in shopping centres, and as such are convenient to their clients:

*“Well I think it’s an excellent service to the mother, drop-in at a shopping centre I think is a very important service, rather than her going to some little building in the middle of woop-woop, where you know there’s no shelter, and if it’s raining they get wet coming from their car to the building.”* RESP 8

Most pharmacy nurses reported high demand for their services, with some reporting extremely busy clinic sessions where they might see up to 25 mothers in a single clinic of three to four hours.

### Services provided by pharmacy nurses

Pharmacy nurses reported two main types of care provision: first, practical and emotional support to parents (particularly mothers), and second, health surveillance of babies. They perceived that they were able to provide meaningful emotional support, reassurance, and empowerment in a flexible, responsive clinic environment:

*“I think that women need somewhere they can go to for support and can come and feel comfortable that someone will listen to them … the most important thing is basically empowering the mothers and basically offering support for them.”* RESP 11

*“…reassurance plays a big part in what I do because sometimes they just want to be told that what they’re doing and the baby…is thriving.”* RESP 6

“ *weighing; a lot of advice and support. I find a big part is just counselling mothers because they're coping with something completely new. To me that's the big thing*.” RESPR1

Infant checks including weight, other measurements and general parenting advice were among the other most frequently cited types of services provided. A much smaller proportion, about one quarter of our sample, reported providing advice and information about infant and other products for purchase.

### Constraints, concerns and limitations

Pharmacy nurses were also asked to comment on any constraints or concerns they had with their practice in pharmacies. The key concerns they listed were professional isolation in their practice, lack of opportunities for professional development, lack of privacy for consultations, inadequate facilities for bare weighing of babies or providing breast feeding advice and support, and ad hoc or non-existent mechanisms for record keeping or client follow up. The nurses themselves and the GPs also provided insight into the existing mechanisms for the integration of care through referrals, and for how these might be improved.

Pharmacy nurses practise on their own, albeit with some contact with the staff of the pharmacies in which they work. They do not work alongside peers, nor do they have back up when they are sick. They do not have a professional association to provide support, peer contact or professional development. As we have seen, most are well qualified in areas relevant to their practice, but many expressed concerns about the difficulties of keeping up to date with developments in practice, especially when informational seminars run by formula companies were amongst the few opportunities available to them.

*“we’re very isolated in the community as nurses so it’s up to myself to keep myself updated with information, what’s the latest and if I’m sick there’s no one to back me up....I try to go to as many seminars as we’re invited to. They would be run by the formula companies and even though they’re sponsored by formula companies, the information they give at seminars are usually by paediatricians or specialists in whatever field may be applicable to us so it’s not about formula. So we get to meet other nurses that way.”* RESPW1

As well as isolation and lack of opportunities for professional development, pharmacy nurses work in a context that poses several other impediments to the provision of high quality care. Most reported lack of facilities and space to undress and assess babies, resulting in the common practice of weighing babies dressed. Similarly, pharmacy nurses reported feeling constrained in their ability to provide mothers with breastfeeding support because of lack of privacy and to some extent the lack of time. One of the pharmacy nurses who is also qualified as a lactation consultant expressed her concerns about this limitation in capacity to support new mothers. This pharmacy nurse also expressed concern about her proximity to infant formulas in the pharmacy, which she felt implicitly endorsed these feeding products.

Another widespread concern was with record taking and keeping. The relatively isolated, autonomous and unregulated practice of pharmacy nurses meant that there is no consistent approach to record keeping. Information collected was quite variable (infant weighs notwithstanding). Whether they kept records at all, and if so what and where, was even more varied. Most respondents indicated limited capacity to store records, and few had access to a computer. One of the interviewees expressed concern that she had no capacity to follow up clients if they did not make a return visit.

At the present time, pharmacy nurse practice is essentially unregulated in Queensland; there is no statutory body that provides oversight or regulation, and pharmacy nurses themselves do not have a professional association. As already discussed, our sample of pharmacy nurses are experienced and well qualified for the work they undertake in pharmacies, but without some form of regulatory oversight by a professional association or statutory body, there is no assurance that all nurses working in pharmacies will be appropriately qualified. This has implications for primary care coordination and integration, as discussed in the next part of the paper.

### Primary care coordination and integration

One of the important indicators of primary care coordination and integration is the existence and use of referral pathways. Very few of our sample of pharmacy nurses - less than one fifth - explicitly mentioned referrals to other health care practitioners as part of their routine practice. They also reported few mechanisms or structures that might facilitate the integration of care with other primary care providers, apart from one exception: in Queensland, all new parents are issued with a child health record ‘book’ (commonly known as the ‘red book’) by the birthing facility at which the birth took place. The book is intended for the documentation of routine health surveillance and immunisations, and provides information about the infant’s birth, weight and condition at birth. If the parents brought the ‘red book’ to the pharmacy, most of our respondents indicated they would refer to it. Some also used it to record infant weight or other measures, or to make notes in the back of the book. However, there was no systematic use of the child health record by pharmacy nurses, and therefore no consistent formal mechanism for communicating information to other primary care providers.

Informal referrals, particularly word of mouth recommendations, were much more commonly used, but patterns of recommendation to or from pharmacy nurses varied substantially, and tended to reflect relationships based on personal knowledge of individuals. This is not surprising given the lack of certainty about the credentials and qualifications of pharmacy nurses. One of the GPs in our sample summarised this clearly by saying

“*Some pharmacies have nurses who do weighs and measures of baby, although that can be hit and miss in terms of what is their training and their background, and certainly some of the advice that women have received from them has been quite inappropriate in my experience, and others of them seem pure gold, so it’s just that you don’t have that control over them, and well my understanding is there isn’t any sort of rigorous system in place to make sure [about] the information they are giving women…”* GPBR1

Of course, these kinds of variations in the quality of care provided can be seen across a range of practitioners; the problem for pharmacy nurses is, as noted earlier, the lack of clear and consistent credentials required for the job, since the occupation is currently unregulated.

Working relations with CFHNs tended to be particularly problematic for different reasons. A number of pharmacy nurses had previously worked in public CFHCs, and were critical of what they described as their bureaucratic, inflexible, unresponsive and intrusive aspects. They rarely recommended the CFHCs, although some informed their clients about specific services at Child and Family Health, such as

“*the little [six week] course for mums if you make your appointment.*” RESPR1

One pharmacy nurse referred mothers to the CFHC because of the limitations of the facilities she had access to at the pharmacy. Some of the limitations included lack of space for private consultations, and inappropriate facilities for bare weighing babies.

Conversely, pharmacy nurses rarely had women referred to them by public CFHCs. Only one pharmacy nurse indicated that some mothers of ‘well babies’ were told of her services by the local CFHC. More frequently, pharmacy nurses spoke of a difficult relationship with public providers of child and family health, and mentioned feeling somewhat denigrated by staff in publicly provided CFHCs. For example, one nurse told us “*I don’t think Community Health would send anybody to us. I’ve had women’s feedback....that [pharmacy nurses] aren’t ‘proper’ Child Health nurses”* RESPR1.

Our respondents were cognisant of their inability to make formal referrals to other health care providers, and would advise women to seek a formal referral from a doctor if necessary. The patterns of relationship and referral with GPs, obstetricians, paediatricians, pharmacists and hospital midwives were quite variable, and depended very much on personal knowledge of particular practitioners. For example, in several cases, pharmacy nurses either worked for GPs, or in clinics with midwives in private hospitals. These relationships, based on personal knowledge and experience of a particular individual, facilitated informal referrals between them. For example,

“....*where I work [in a General Practice], particularly one of the doctors - initially I just worked for her - she would certainly tell women go and see me. I think when they know you [they refer on to you*].” RESPR 1

On the other hand, when these relationships were not present, GPs expressed concerns about ensuring consistency in the quality of care offered by pharmacy nurses as noted above.

Most of the pharmacy nurses indicated that they would informally suggest that parents seek the advice of a GP where necessary, although some expressed hesitation because of the time and cost involved in seeing a doctor. In addition, decisions about ‘necessity’ are potentially subjective, and may be dealt with differently depending on the health care provider involved, as the following interview excerpt demonstrates:

Interviewee: But the people who need - they need to talk to you about something - then they are going to persist. Because what are they going to do? Go and sit down at a doctor's surgery - because some doctors take an hour-and-a-half to see you? Then they've got to pay for that too, don't they?

Facilitator: Yes.

Interviewee: So that side of things - as an alternative to a doctor's advice. I do get some people who say, they've got this and this - what do you reckon?

Facilitator: That puts you in an awkward position.

Interviewee: It does but a lot of it's basic stuff. You know, if they have a bit of a temperature and viral things I will say, this is how viruses work, this is what happens. But again you've got to say, if you are worried, as you know, you go to the doctor.

So a lot of it's just basic little things. A little bit of stuff gets a bit hard because you can buy cortisone over the counter, you can buy DermAid. With some of those little dermatitises, I know very well they're going to go to the doctor and that's exactly what they're going to give them and they're going to pay $65 for a consult and that. For some people, I will tell them they can have that over the counter if they want. Sometimes I'll take them and get the pharmacist to have a look at stuff and see if they're happy to recommend something they can buy over the counter. Sometimes they won't, they can't give them something over the counter. So I do use the pharmacist as well.

The direct referral to a pharmacist noted above was not mentioned often by our respondents, but it is important in its implications for the provision of primary health care, especially if pharmacy nurses feel pressured to promote products within the pharmacy. Only one of our respondents indicated that she had been asked to do this. All said that they would not promote products simply to achieve a sale, although four of the pharmacy nurses said they promoted products because they believed in the quality of the product sold by the pharmacy. Overall the nurses were positive about the pharmacists who employed them and respected their professional integrity. All nurses explicitly referred to the fact that endorsing products was not a formal condition of their employment. However, more subtle implicit pressure, expressed by one nurse as the need to ‘cover my wage’, was clearly a concern for some pharmacy nurses.

Overall, coordination with other primary care providers of postnatal or early parenting care was ad hoc and variable at best. Several of the pharmacy nurses referred clients to lactation consultants, and some older infants were referred to allied health professionals (physiotherapists or podiatrists). In sum, the views of our sample about working with other primary care providers indicated that few used systematic channels for referring to GPs, pharmacists, or CFHCs, and some interviewees recounted a ‘hostile’ relationship between the CFHCs and the pharmacy nurse services. Apart from particularistic relationships with employing pharmacists or GPs, pharmacy nurses generally practise in isolation, with no systematic avenues or mechanisms for service coordination or integration with other primary care providers.

## Discussion

The pharmacy nurse model described in this paper appears to be unique to Australia, and even within Australia exists in only some States. In Queensland, pharmacy nurses who provide maternal and child health services do so free of charge to their clients, unlike the well-baby services in North America which operate on the basis of a fee-for-service, and unlike the publically funded Health Visiting Service in the United Kingdom [13,14].

Prior to this study, little was known about the attributes or motivations of pharmacy nurses in Queensland [6]. The pharmacy nurses in this study were highly qualified and experienced in fields relevant to maternal and child health. Many had worked in publically funded child and family health services prior to working in pharmacies. They were attracted to pharmacy work for a mixture of personal reasons such as predictable hours compatible with family responsibilities, as well as intrinsic features of their work practice. These included considerable autonomy and freedom from the bureaucratic constraints some spoke of in the public sector. In addition, many felt they had the capacity to provide woman-centred, responsive care that was accessible and conveniently located. These findings suggest that the concerns of some GPs about the possible lack of consistent minimum, professional qualifications could be allayed [8,10]. However, further research with a larger, representative sample of nurses would be needed to determine this.

The sample of pharmacy nurses interviewed for this study were all paid by the employing pharmacy, mostly as casual employees. This study did not explore the reasons pharmacy owners have engaged the services of nurses to provide maternal and child health care, but consumer demand for the services is evident in the large volume of clients, and possibly these clients bring with them the potential for pharmacy sales. Nonetheless, the nurses interviewed did not feel pressured to endorse or promote products. A minority expressed their own perceived concerns about ‘covering’ their wages; and in a few cases nurses felt their location near baby products rendered a subtle, though not explicit, endorsement of the products on sale.

Pharmacy nurses offer a suite of services similar to, though more limited than, those provided through the publically funded child and family health service in Queensland, which operates primarily through CFHCs. These services included weighing of infants, parenting advice, reassurance and health promotion activities [6]. While this study has not explored the historical emergence of pharmacy baby clinics, the nurses interviewed shared the widely held view that pharmacy based clinics have emerged in response to the reduction and realignment of publically provided child and family health services in Queensland [24]. Both pharmacy nurses and GPs involved in this study shared the view that the public system of care is overstretched, and unable to meet the needs of early parenting families. Many of the nurses interviewed had worked in the public system, and all were familiar with it. A majority of nurses reflected on the reduction of publically provided care, especially in terms of the increased centralization of CFHCs, as well as the increased emphasis on targeted rather than universal services. Even though pharmacy nurses offer a more limited range of services than CFHCs (for example, they do not offer parenting classes, developmental assessment, mothers’ groups, immunization and other programs), they are more numerous and geographically spread in some areas, particularly the BCC, and hence more accessible than their public counterparts [22]. In addition, drop-in rather than appointment based practices offered access for mothers on a more flexible basis than CFHCs. From the perspective of pharmacy nurses themselves, some of the constraints on their practice included limited physical space for privacy and record keeping, and, importantly, the relative isolation and limited opportunities for professional development.

From the perspective of health care services more broadly, the provision of care by pharmacy nurses has emerged on an ad hoc basis as a market driven response to gaps in the public sector. As such it is not surprising that there is little or no formal connection between the two sectors, potentially exacerbating known issues with poor service coordination and integration [11,20,21]. While there is clearly demand for both in Queensland, the lack of coordination between them requires further examination for its impact on mothers and their families.

### Limitations

This study provides important preliminary findings about an under-researched form of maternal and child health care provision in Australia. The study provides new knowledge about the prevalence of pharmacy clinics within the BCC. However, little is known about their prevalence in the remainder of the State, or in other parts of Australia. Descriptive data which could provide a more definitive picture of the prevalence of pharmacy based baby clinics is needed to contribute to an understanding of this form of care in the context of publically funded child and family health nursing services.

One of the major limitations of the study is its reliance on a small number of pharmacy nurses and GPs. The interviews comprise a small scale, qualitative study. A larger study which could establish the representativeness of the sample to the population would further contribute to the body of knowledge in this area.

Another limitation is its reliance on only two types of health care providers. The inclusion of other key stakeholders in any further research would be an important next step. The insights and experiences of pharmacy owners, employees in the public sector providing community maternal and child health care, and newly parenting mothers in Queensland would contribute to a fuller understanding of the genesis and role of pharmacy nurse clinics, and what it means for the overall provision of care.

## Conclusions

The prevalence of pharmacy based clinics in Queensland appears to be a market-driven response to gaps in the public provision of maternal and child health services. The pharmacy nurses at these clinics report high levels of demand for their services, which focus on maternal reassurance, support, infant weighing and monitoring. Pharmacy based clinics provide higher levels of access than CFHCs, convenience and choice to parents, as well as continuity of carer. The large number of pharmacy clinics relative to publicly provided CFHCs in some geographic areas offer several distinctive advantages. They are primarily drop-in services conveniently located in terms of access to retail outlets and other services. As with many health care professional contacts, the quality of care provided is largely dependent on individual attributes and the autonomous practice of particular nurses. As a group, our sample of pharmacy nurses are relatively highly trained and experienced, but, unlike the majority of nurses in Australia, their work is not overseen by their employer or any other professional organisation, nor are they bound by guidelines, protocols and procedures, or have clearly articulated referral pathways, an integral part of most other health care settings. While they are able to offer responsive, client focused care, they have limited opportunities for updating skills and other forms of professional development, including contact with peers. Importantly, because their practice tends to be isolated, they report little formal or informal connection with other primary health care services, exacerbating problems with lack of service coordination and integration in primary health care services more generally. While providing an accessible support service, with continuity of carer, the current arrangements for nurses practising in pharmacies could be improved by increasing the availability of professional development activities and interaction with other nurses working in the field. In addition, the provision of appropriate consultation facilities, including privacy for the mother and infant, would enhance the ability to deliver high quality, best-practice care.

## Abbreviations

CFHN: Child and Family Health Nurse; CFHC: Child and Family Health Centre; GP: General Practitioner; BCC: Brisbane City Council; UQ: University of Queensland; UPNCS: Universal Postnatal Contact Service.

## Competing interests

The authors declare that they have no competing interests.

## Authors’ contributions

MZ worked with the co-authors in designing the study, conducted interviews, analysed data and prepared the manuscript. WB assisted in the design of the study, conducted interviews, analysed data and assisted in the preparation of the manuscript. LF, LP and CN assisted in the design and implementation of the mapping of the prevalence of pharmacy nurse clinics in the BCC, conducted interviews, analysed data and assisted in the preparation of the manuscript. SK assisted with preparation of the manuscript. All authors read and approved the final manuscript.

## Pre-publication history

The pre-publication history for this paper can be accessed here:

http://www.biomedcentral.com/1471-2393/13/144/prepub
